# Influence of Carving Ski Base Preparation on Patient-Reported Outcomes in Recreational Skiers with Knee Osteoarthritis: A Pilot Study

**DOI:** 10.3390/bioengineering13070772

**Published:** 2026-07-01

**Authors:** Bianca Valentina Schlesier, Christian Soost, Jan Adriaan Graw, Rene Burchard, Artur Barsumyan

**Affiliations:** 1 Faculty of Medicine, Philipps-University of Marburg, 35032 Marburg, Germany; 2 Faculty III: Statistic and Econometrics, University of Siegen, 57076 Siegen, Germany; 3 Department of Anaesthesiology, Friedrich-Alexander-Universität Erlangen-Nürnberg (FAU), Universitätsklinikum Erlangen, 91054 Erlangen, Germany; 4 Department of Orthopedics and Traumatology, University Hospital of Giessen and Marburg, 35043 Marburg, Germany; 5 Sports Medicine and Joint Centre, Department of Orthopedics and Trauma Surgery, Lahn-Dill-Kliniken, Rotebergstr. 2, 35683 Dillenburg, Germany

**Keywords:** winter sports, gonarthrosis, IKDC, carving on-piste

## Abstract

Introduction: For many people with knee osteoarthritis, alpine skiing is an important form of physical activity that improves their quality of life. However, pain and perceived instability can limit participation. While it is recognized that ski preparation affects performance, the impact of specific gliding surface preparation on pain and functional stability in patients with knee osteoarthritis has not yet been scientifically examined. This pilot study examined whether preparing the base of carving skis professionally could alleviate subjective symptoms and improve knee function while skiing. Methods: Six patients (*n* = 6) with clinically diagnosed gonarthrosis were included in the uncontrolled pilot study. Before and after the targeted ski preparation intervention, the participants completed standardized patient-reported outcome measures, using the International Knee Documentation Committee score. The intervention involved optimizing the preparation of the ski gliding surfaces and edges. Pre- and post-intervention scores were statistically compared to assess changes in pain, function, and perceived stability. Results: Targeted ski base preparation was associated with improved patient-reported pain and subjective stability while skiing. Functional outcome measures also improved after the intervention. The International Knee Documentation Committee (IKDC) score increased from 58.54 ± 17.66% pre-intervention to 85.37 ± 14.95% post-intervention (*p* = 0.043). The estimated median paired difference was 31.7% (95% CI, 7.3% to 45.1%), indicating enhanced knee function after the intervention. This corresponded to a large effect size (rank-biserial r = 0.86) and exceeded the minimal clinically important difference for the IKDC, with five of six participants improving and none deteriorating. Conclusions: The professional and targeted preparation of carving ski gliding surfaces was associated with improvements in patient-reported pain and functional stability in this exploratory pilot study. Given the uncontrolled design and small sample, these observations should be regarded as hypothesis-generating and require confirmation in adequately powered, controlled trials. These findings highlight the potential value of sport-specific equipment adaptations as a complementary approach to conventional therapies. Further studies with larger sample sizes are required to confirm these results and evaluate the long-term effects.

## 1. Introduction

With an estimated 40–50 million active participants, alpine skiing is one of the most popular winter sports in Europe [1]. Regardless of age, sex or skill level, skiing offers enjoyment, physical activity and psychological benefits. However, alpine skiing also involves inherent injury risks and requires a certain level of technical competence. Following knee injuries or degenerative joint diseases, many individuals report uncertainty about returning to skiing at all or experience pain and instability in the knee joint [2].

For patients with knee osteoarthritis (gonarthrosis), alpine skiing can become more painful and feel less stable than before the onset of the disease [3]. Nevertheless, giving up skiing after a diagnosis of gonarthrosis is inconceivable for many individuals, because the sport represents an important part of their lifestyle and quality of life [4]. Several studies have examined the possibility of skiing after a total knee arthroplasty, the definitive treatment for advanced knee osteoarthritis [5,6,7]. There is growing recognition that regular physical activity and continued sports participation are beneficial rather than harmful for most individuals with knee osteoarthritis, supporting strategies that enable patients to remain active [8].

Carving is a skiing technique in which the skis are angled onto their edges to cut clean arcs through the snow, leaving distinct tracks. In contrast, skidding leaves wider trails. The carving technique offers distinct advantages in terms of skiing mechanics and comfort on the piste [9]. According to Dingerkus and Mang [10], the shorter length and pronounced sidecut of carving skis enable more efficient turning with reduced effort. Skiers of all ages and abilities describe the experience of carving as controlled, smooth and effortless [11]. Concerns that the carving technique might increase joint loading have not been substantiated so far [12]. The carving technique has been described as comparatively ‘joint-friendly’. Biomechanical comparisons of carved and skidded turns lend partial support to this characterization: Klous et al. reported markedly lower peak knee extension moments in carved than in skidded turns (4.07 vs. 8.35 Nm·kg^−1^), suggesting reduced sagittal-plane joint demand during carving [13], a line of work extended by three-dimensional analyses of lower-extremity loading across turn types [14]. Lower muscular activation and greater conservation of force reserve relative to traditional parallel technique have likewise been described [15]. The evidence is not uniform, however: carved turns are typically executed at higher speeds and edge angles and can produce comparable or greater frontal-plane (adduction) knee moments and ground-reaction forces [13]. The ‘joint-friendly’ designation should therefore be regarded as technique- and phase-dependent rather than absolute and warrants confirmation in osteoarthritic populations specifically.

The preparation of the gliding surface of the ski (brushing, cleaning and waxing) could significantly influence skiing performance and the smoothness of movement [16]. A study by Heinrich shows that unwaxed ski bases and poorly sharpened edges cause greater motor strain for skiers [17]. Despite extensive work on ski preparation in the context of performance, a clear gap remains: no study has examined whether such preparation influences pain or perceived knee stability in patients with knee osteoarthritis [15,18]. The objective of this pilot study was therefore to determine whether professional, targeted preparation of carving-ski gliding surfaces and edges is associated with measurable changes in patient-reported knee symptoms (IKDC) in recreational skiers with gonarthrosis. The novelty of the present work lies in transferring a question previously confined to skiing performance in healthy and elite athletes into a clinical population: it is, to our knowledge, the first study to quantify whether targeted equipment preparation can modify patient-reported knee symptoms in individuals with gonarthrosis.

This study aimed to investigate the influence of ski gliding surface preparation on pain perception and subjective knee stability in patients with knee osteoarthritis during on-piste carving. The study focused on recreational alpine skiing on groomed slopes rather than off-piste skiing or competitive racing. By comparing pain perception and perceived stability before and after ski preparation, the study sought to improve understanding of whether ski equipment preparation could positively influence the skiing experience of patients with gonarthrosis. Specifically, the study examined whether patients with gonarthrosis experienced pain or functional limitations in daily life, whether targeted ski base preparation affected their ability to ski, and whether such preparation might improve International Knee Documentation Committee (IKDC) scores. In addition, the study examined whether patients with knee osteoarthritis who experienced pain during daily activities were able to resume skiing following ski preparation.

## 2. Materials and Methods

Pilot Study Reporting Statement: This investigation was designed as an exploratory pilot study to evaluate feasibility, preliminary effect estimates, and methodological considerations for future adequately powered trials. In accordance with CONSORT extension guidelines for pilot and feasibility trials, no formal sample size calculation was performed [19]. Accordingly, the present sample (*n* = 6) is not intended to provide definitive efficacy estimates but to assess recruitment feasibility, protocol acceptability, and the plausible direction and magnitude of effect for the design of a subsequent randomized controlled trial. All inferential results are therefore reported as preliminary effect estimates with their associated uncertainty, rather than as confirmatory findings.

Eligible participants were active alpine skiers with a confirmed clinical diagnosis of knee osteoarthritis (gonarthrosis), presenting at the sports orthopedic outpatient clinic between April 2020 and April 2023. Only patients who reported regular participation in recreational skiing were considered for inclusion. Knee joint status was categorized using radiographic osteoarthritis grading according to the Kellgren–Lawrence scale [20,21]. 

This study was conducted in accordance with the Declaration of Helsinki (2013), and the ethics committee of the Philipps-University of Marburg approved the study (23–130 RS). The study was not prospectively registered, as it was conducted as an exploratory, hypothesis-generating pilot investigation without a confirmatory efficacy aim.

### 2.1. Patient-Reported Outcome Measures

Patient-reported outcome measures (PROMs) were used to assess pain, knee function, and subjective knee stability in patients with gonarthrosis. For this purpose, we used the International Knee Documentation Committee (IKDC) score [22]. This score was subsequently used to compare patient-reported outcomes between the different ski gliding surface preparation conditions.

PROMs are widely used to assess joint function and symptom burden in patients with knee osteoarthritis and are internationally validated in surgical and non-surgical clinical settings [23]. To quantify the effect of ski base preparation on pain and functional perception during skiing, the IKDC score was used. The IKDC subjective knee form was administered with the activity-reference frame contextualized to on-piste recreational skiing: the standard symptom and function items were retained without altering their wording or scoring algorithm, while participants were instructed to rate symptoms specifically during and after carving rather than during everyday activity. No items were added or removed, and the original 0–100 scoring metric was preserved. As this represents a contextual administration rather than a re-validated instrument, the adapted use is treated as a methodological limitation.

### 2.2. Statistical Analysis

Due to the small sample size and potential deviations from normality, paired differences between pre- and post-measurements were analyzed using the Wilcoxon signed-rank test. Results are reported with corresponding *p*-values and bootstrap confidence intervals for the median difference (Hodges–Lehmann estimator). The primary outcome was the change in IKDC score (post–pre) following ski base preparation. Continuous data are reported as mean ± standard deviation (SD). Post hoc effect sizes (rank-biserial correlation) with corresponding bootstrap confidence intervals were additionally calculated to provide an estimate of the magnitude and precision of the observed effects. Given the exploratory nature of the study and the small sample size, the focus was placed on confidence intervals, as these reflect both the direction and the uncertainty of the effect estimate and thus provide a more informative basis for interpretation than isolated effect sizes. A significance level of α = 0.05 was applied. Analyses were performed in R (Version 4.5.3).

### 2.3. Ski Preparation

**Figure 1:**
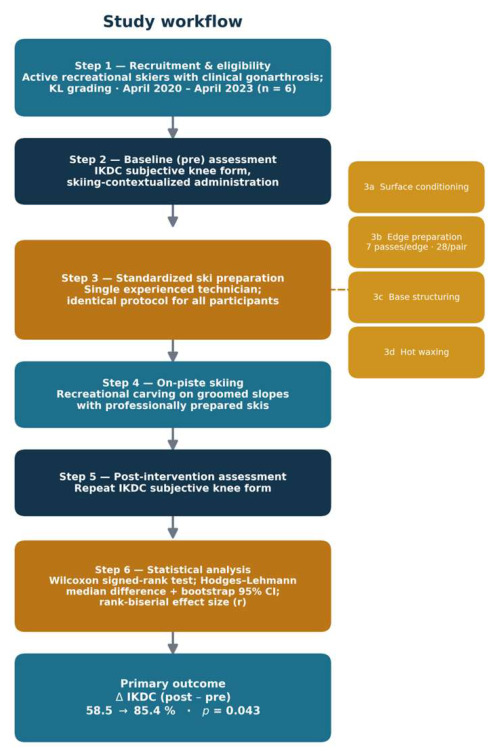
Step-by-step workflow of the study design and standardized ski-preparation protocol.

Ski base preparation is a multi-step process involving adjusting the skis according to the skier’s technique, the snow conditions and other external factors [24]. All participants in this study received professional ski preparation performed by an experienced technician. This included initial surface conditioning, systematic edge preparation, base structuring and waxing (Figure 1).

**Figure 2:**
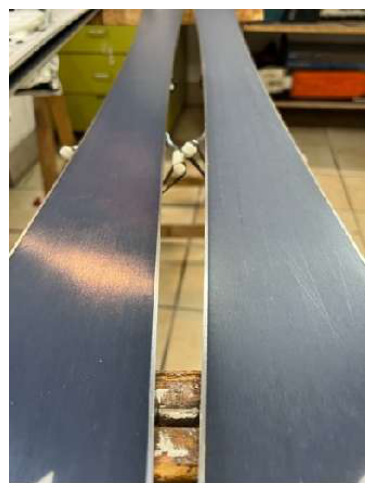
Optical comparison between a prepared ski base (**left**) and an unpolished ski base (**right**). The prepared ski shows a uniform grinding microstructure with a smoother and more reflective surface, whereas the unpolished ski exhibits a rougher texture with irregularities and reduced surface uniformity.

Edge preparation was standardized and consisted of seven grinding passes per edge. As each ski has two edges, this resulted in 14 grinding passes per ski and 28 passes per pair. The identical standardized protocol was applied to all participants by the same experienced technician to minimize preparation variability. Figure 2 illustrates the visual outcome of the preparation process, showing a prepared ski (left) in comparison with an unpolished ski (right).

The preparation effect is documented only qualitatively (Figure 2); no quantitative surface investigation (e.g., roughness parameters Ra/Rz by profilometry) was performed to characterize the prepared versus unprepared bases.

## 3. Results

Table 1 summarizes the participant (*n* = 6) demographic characteristics, including age, sex, height, weight, self-reported skiing ability and radiographic grade of knee osteoarthritis.

**Figure 3:**
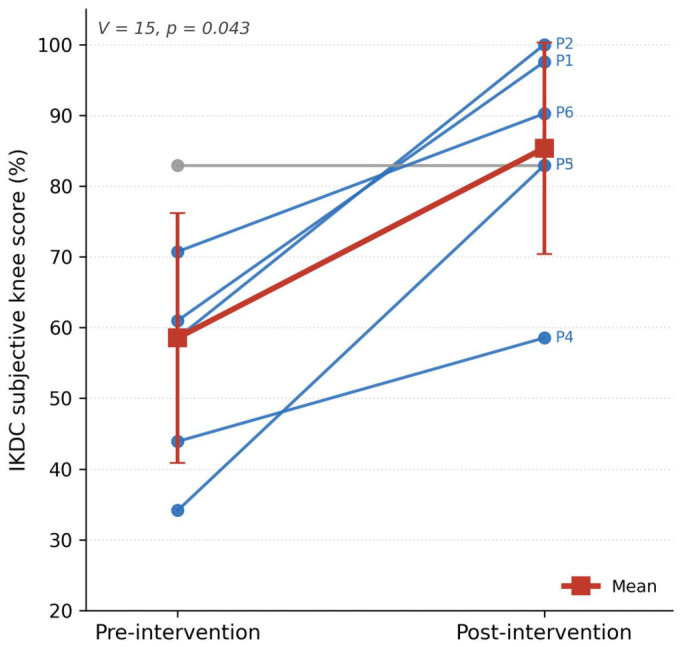
Individual pre- and post-intervention IKDC subjective knee scores (*n* = 6). Each line represents one participant; the grey line denotes the single unchanged participant. The red marker shows the group mean ± SD. Wilcoxon signed-rank test: V = 15, *p* = 0.043.

IKDC scores increased from 58.54 ± 17.66% (pre) to 85.37 ± 14.95% (post), corresponding to a mean paired change of 26.83 percentage points. The Wilcoxon signed-rank test showed a statistically significant increase in scores after the intervention (V = 15, *p* = 0.043). The estimated median paired difference was 31.7% with a 95% bootstrap confidence interval ranging from 7.3% to 45.1%. The post hoc effect size analysis indicated a large effect (r = 0.86, 95% CI [0.7, 0.92]) for the change in IKDC scores from pre- to post-measurement (Figure 3). The robust effect size estimates should be interpreted with caution given the small sample size and the exploratory nature of the study.

## 4. Discussion

Consistent with the exploratory nature of a pilot study, we interpret the findings in terms of feasibility, and the direction and precision of effect estimates rather than statistical significance. The observed effect magnitudes are compatible with a potentially meaningful functional benefit, but the wide confidence intervals—reflecting limited statistical power—and the absence of a control condition mean that the strength of evidence remains low and that confirmatory randomized controlled trials are required. The present findings should therefore be interpreted as proof-of-concept evidence rather than definitive efficacy data.

This pilot study examined how targeted preparation of the base and edges of downhill skis, particularly carving skis, affected pain perception and subjective knee stability in patients with knee osteoarthritis. The study’s primary finding is that professional, targeted preparation of the ski gliding surfaces was associated with a significant reduction in pain and a noticeable improvement in perceived stability while skiing.

As Sørensen et al. have noted, no single PROM can be considered universally superior in the management of knee osteoarthritis; rather, different instruments provide complementary and clinically relevant information [25]. PROMs revealed meaningful improvements in functional parameters following the intervention. Having control over the skis is important for skiers. Prepared edges enable skiers to control the skis more precisely, which increases the feeling of stability and reduces the risk of falling [26]. This greater sense of safety while skiing could be due to the increased edge grip and the resulting direct power transmission. The characteristics of carving skis play an important role here. As Kober and Held described, carving skis are comfortable to ride because their pronounced sidecut enables stable edge engagement and allows turns to be initiated with less rotational effort and skidding [12].

Notably, the IKDC score showed a statistically significant increase from pre- to post-intervention, indicating subjectively improved knee joint function. These results imply that relatively straightforward technical adjustments to ski equipment could significantly impact how patients with gonarthrosis perceive their symptoms and how confident they feel in their ability to perform functional activities.

To contextualize the magnitude of the observed change, the mean IKDC improvement of 26.8 percentage points was benchmarked against the minimal clinically important difference (MCID) established for the instrument. Using an anchor-based method, Greco et al. reported an MCID of 16.7 points for the IKDC Subjective Knee Form [27], whereas anchor-based estimates derived specifically in knee osteoarthritis populations vary considerably with the calculation method, ranging from approximately 6.3 to 25.9 points [28]. The mean improvement in the present sample exceeded the canonical 16.7-point threshold. At the individual level, four of the six participants surpassed this threshold and a fifth exceeded the lower anchor-based estimate of 6.3 points, while one participant showed no change. Taken together, these comparisons indicate that the observed improvement is not only statistically detectable but also of a magnitude likely to be clinically relevant for most participants. This interpretation must nonetheless remain cautious: the uncontrolled design, the wide confidence interval, and the well-documented contribution of contextual effects in osteoarthritis (see below) preclude attributing the change in full to the intervention.

A further consideration when interpreting the magnitude of the observed change is the well-documented contribution of contextual effects to symptom improvement in osteoarthritis. In a meta-analysis of 215 randomized controlled trials, Zou and colleagues estimated that approximately 75% of the pain reduction observed across diverse osteoarthritis treatments was attributable to contextual rather than treatment-specific effects [29]. In the present open-label, single-arm setting—where participants knowingly received a professional preparation intervention—such contextual mechanisms, including expectation, increased confidence and the attention associated with the procedure, are likely to account for a substantial proportion of the reported improvement. This reinforces our interpretation of the findings as hypothesis-generating and underscores the necessity of a blinded, controlled comparison to isolate any preparation-specific effect.

The following mechanistic considerations are hypothesis-generating: as no biomechanical data were collected, the proposed pathways remain speculative. From a mechanistic perspective, optimizing base and edge preparation can reduce sudden changes in friction, improve edge grip and enable smoother, more controlled turns [30]. These effects could decrease peak joint loading, reduce compensatory muscle activation and improve neuromuscular control, thereby reducing pain and enhancing stability [31]. These assumptions align with previous biomechanical observations that describe carving skis as ‘joint-friendly’ with lower mechanical demands and reduced muscular strain compared to traditional skis [32].

The results of this study suggest that optimizing sport-specific equipment may represent an additional non-invasive therapeutic approach that could complement conventional treatments such as physiotherapy and analgesic management. Although previous research has shown that preparing skis can substantially influence performance, this is the first study, to our knowledge, to examine specifically the effects of preparing the gliding surfaces of skis in a population with knee osteoarthritis [33]. 

Our findings can also be situated within the broader literature on the resumption of alpine skiing after major knee pathology. Studies on skiing following total knee arthroplasty have demonstrated that, with appropriate adaptation, patients can return to recreational skiing with preserved knee function and good subjective tolerance [4,6], and expert recommendations increasingly support carefully selected return to low-impact alpine skiing rather than blanket avoidance [7]. The present results extend this line of work upstream of arthroplasty—to patients with symptomatic but joint-preserving osteoarthritis—and suggest that, in addition to surgical and rehabilitative measures, optimization of the equipment itself may contribute to a more comfortable and confident skiing experience. Positioning equipment preparation alongside these established adaptation strategies may help patients with gonarthrosis remain engaged in a valued recreational activity for longer.

### Limitations

This pilot study is limited by its small sample size (*n* = 6), which restricts its generalisability and increases the risk of selection bias. The absence of a control condition precludes causal inference. Furthermore, multiplicity of outcomes increases type I error risk. Although non-parametric and bootstrap procedures were implemented, statistical uncertainty remains substantial. Future studies should employ randomized crossover designs with biomechanical outcome measures to substantiate mechanistic interpretations. Additional statistical limitation: With *n* = 6, distributional assumptions are difficult to verify; therefore, non-parametric analyses and bootstrap uncertainty estimates were used to complement the paired *t*-test. For IKDC analysis, Wilcoxon testing required asymptotic approximation due to zero differences and very small sample size (*n* = 6). Furthermore, outcomes were based on subjective PROMs without complementary, objective assessments such as biomechanical or imaging analyses. A specific measurement limitation concerns the outcome instrument itself: although the wording and 0–100 scoring algorithm of the IKDC subjective knee form were preserved unaltered, its administration with a skiing-specific activity-reference frame has not been independently validated. The reliability, responsiveness and measurement properties of this contextual application therefore remain unestablished, and the scores should be interpreted as indicative rather than as output of a formally validated skiing-specific instrument. Future work should include psychometric validation of any sport-contextualized PROM before it is used as a primary endpoint. Several potential confounders were not controlled and may have influenced the results, including snow conditions and temperature, slope difficulty, fatigue, between-participant differences in ski equipment, prior skiing experience and technical level, and day-to-day variation in osteoarthritis symptoms. Because all assessments were performed under field conditions without standardization of these factors, their individual contributions cannot be separated from the intervention effect. Future prospective studies involving larger cohorts, objective biomechanical assessments and longitudinal follow-up are required to elucidate the magnitude and durability of these effects. Causal efficacy cannot be inferred from the present uncontrolled pilot design and remains to be established in controlled trials.

## 5. Conclusions

In conclusion, the targeted and professional preparation of carving ski gliding surfaces was associated with improvements in patient-reported pain and perceived stability in patients with knee osteoarthritis during recreational alpine skiing. Causal efficacy cannot be inferred from the present uncontrolled pilot design and remains to be established in controlled trials. These results highlight the importance of personalized, sports-specific interventions to help individuals with degenerative knee joint disease maintain physical activity, safety and quality of life. Further research is required to define the most effective preparation strategies and evaluate their long-term clinical relevance.

## Figures and Tables

**Table 1:** Demographic characteristics of patients (*n* = 6).

Variable	Category	Value Mean (±SD or %)
Age (years)		59.5 (±7.92)
Height (cm)		172.5 (±8.80)
Weight (kg)		81.5 (±8.50)
Gender	Male	2 (33.3%)
	Female	4 (66.7%)
Ski skill	Advanced	3 (50.0%)
	Superior	2 (33.3%)
	Medium	1 (16.7%)
	Beginner	0 (0%)
Arthrosis	0	1 (16.7%)
	1	1 (16.7%)
	2	2 (33.3%)
	3	2 (33.3%)
	4	0 (0%)

## Data Availability

Data are available on reasonable request to the corresponding authors.

## Abbreviations

The following abbreviations are used in this manuscript:

CONSORT	Consolidated Standards of Reporting Trials
PROM	Patient-reported outcome measures
IKDC	International Knee Documentation Committee
